# Antinociceptive Activity of *Borreria verticillata*: *In vivo* and *In silico* Studies

**DOI:** 10.3389/fphar.2017.00283

**Published:** 2017-05-22

**Authors:** Rosa H. M. Silva, Nathália de Fátima M. Lima, Alberto J. O. Lopes, Cleydlenne C. Vasconcelos, José W. C. de Mesquita, Ludmilla S. S. de Mesquita, Fernando C. V. M. Lima, Maria N. de S. Ribeiro, Ricardo M. Ramos, Maria do Socorro de S. Cartágenes, João B. S. Garcia

**Affiliations:** ^1^Experimental Study of Pain Laboratory, Department of Physiological Sciences, Federal University of MaranhãoSão Luís, Brazil; ^2^Laboratory of Pharmacognosy, Department of Pharmacy, Federal University of MaranhãoSão Luís, Brazil; ^3^Research Laboratory Information Systems, Department of Information, Environment, Health and Food Production, Federal Institute of PiauíTeresina, Brazil; ^4^Experimental Study of Pain Laboratory, Department of Pain and Palliative Care, Federal University of MaranhãoSão Luís, Brazil

**Keywords:** *Borreria verticillata*, COX-2, NMDA receptor, molecular docking, molecular dynamics simulations

## Abstract

*Borreria verticillata* (L.) G. Mey. known vassourinha has antibacterial, antimalarial, hepatoprotective, antioxidative, analgesic, and anti-inflammatory, however, its antinociceptive action requires further studies. Aim of the study evaluated the antinociceptive activity of *B. verticillata* hydroalcoholic extract (EHBv) and ethyl acetate fraction (FAc) by *in vivo* and *in silico* studies. *In vivo* assessment included the paw edema test, writhing test, formalin test and tail flick test. Wistar rats and Swiss mice were divided into 6 groups and given the following treatments oral: 0.9% NaCl control group (CTRL), 10 mg/kg memantine (MEM), 10 mg/kg indomethacin (INDO), 500 mg/kg EHBv (EHBv 500), 25 mg/kg FAc (FAc 25) and 50 mg/kg FAc (FAc 50). EHBv, FAc 25 and 50 treatments exhibited anti-edematous and peripheral antinociceptive effects. For *in silico* assessment, compounds identified in FAc were subjected to molecular docking with COX-2, GluN1a and GluN2B. Ursolic acid (UA) was the compound with best affinity parameters (binding energy and inhibition constant) for COX-2, GluN1a, GluN2B, and was selected for further analysis with molecular dynamics (MD) simulations. In MD simulations, UA exhibited highly frequent interactions with residues Arg120 and Glu524 in the COX-2 active site and NMDA, whereby it might prevent COX-2 and NMDA receptor activation. Treatment with UA 10 mg/Kg showed peripheral and central antinociceptive effect. The antinociceptive effect of *B. verticillata* might be predominantly attributed to peripheral actions, including the participation of anti-inflammatory components. Ursolic acid is the main active component and seems to be a promising source of COX-2 inhibitors and NMDA receptor antagonists.

## Introduction

Pain is a warning system that informs the body about the occurrence of tissue damage (Nickel et al., 2012). In the pathophysiology of pain several biological actions are involved, including activation of cyclooxygenase 2 enzyme (COX-2) and N-methyl-D-aspartate (NMDA) receptor.

COX-2 is upregulated in the central nervous system in response to inflammatory factors. It is a rate-limiting enzyme for prostanoid production during inflammation (Ricciotti and Fitzgerald, 2011). Prostaglandin E2, the main pro-inflammatory prostanoid, induces painful hypersensitivity through modulation in the nociceptive pathways, activates the periphery ionic channels such as sodium, calcium, and potentiates the central activation of NMDA and α-amino-3-hydroxy-5-methylsoxazol-4-propionic (AMPA) receptors (Chen et al., 2013). Inhibition of COX-2 enzymatic activity prevents prostanoid production, thus this enzyme is a usual target of non-steroidal anti-inflammatory drugs (NSAIDs) (Zaiss et al., 2014).

The activation of NMDA receptor requires the binding of glycine and glutamate to its subunits GluN1 and GluN2, respectively (Tajima et al., 2016). It is well-known that activation of NMDA receptors causes central sensitization, amplification of spinal nociception, increased ionic conductance and membrane depolarization (Phang and Tan, 2013). For this reason, NMDA receptor antagonists (e.g., memantine) are considered an option in the management of opioid-resistant and chronic pain (Hewitt, 2000).

Therefore, NSAIDs and NMDA receptor antagonists are used to afford pain relief. However, the use of these agents is limited by the occurrence of side effects, such as dizziness, vomiting, constipation, and gastric erosions. These problems and the impact of pain in the quality of life of patients evidence the need of novel therapeutic targets for pain management.

Medicinal plants and their derivatives represent a common alternative for the treatment of diseases (Kandimalla et al., 2016; Zaia et al., 2016). *Borreria verticillata* (L.) G. Mey., known in Brazil as poaia, cordão-de-frade and vassourinha (Júnior et al., 2012) is traditionally used for various therapeutic purposes including the treatment of pain and inflammatory conditions (Vieira et al., 1999; Souza et al., 2013). It has shown to possess antibacterial (Neto et al., 2002; Ogunwande et al., 2010; Balde et al., 2015), hepatoprotective (Murtala et al., 2015), antioxidant (Abdullahi-Gero et al., 2014a), anti-inflammatory and analgesic (Abdullahi-Gero et al., 2014b) activity.

New technologies have been applied to the assessment of the pharmacological properties of extracts and active principles of medicinal plants, such as molecular docking and molecular dynamic, which is a computer-based approach used to give a prediction of the ligand-receptor complex structure (Meng et al., 2011). The combination of computational technique with biological assay became an important strategy toward finding plant-based drugs (Sharma and Sarkar, 2012).

Considering the factors that contribute to the mechanisms of pain and the use of medicinal plants as multi-targets therapeutic alternatives, the aim of the present study was to assess the antinociceptive activity of the crude hydroalcoholic extract and ethyl acetate fraction of *B. verticillata*. Furthermore, evaluate the molecular interactions of compounds present in ethyl acetate fraction with COX-2 enzyme and NMDA receptor.

## Materials and methods

### Botanic material

The aerial parts of *Borreria verticillata* (L.) G. Mey, Rubiaceae were collected at São José de Ribamar, Maranhão state, (2°33′13.3″ S 44°11′22.8″ W), Brazil, in July 2014. A voucher specimen was deposited at Maranhão Herbarium (MAR), of Federal University of Maranhão (UFMA), under the registration number 5151.

### Obtaining the hydroalcoholic extract and the ethyl acetate fraction

Aerial parts of *B. verticillata* were dried at 38°C in an oven with circulating air and powdered with a knife mill to obtain a moderately coarse powder (particle sizes under 710 μm and over 250 μm). The powder of *B. verticillata* aerial parts was macerated with 70% ethanol for 5 days (this step was repeated 3 times) obtaining a solution. The solution was filtered and concentrated to a small volume at 40°C in a rotary evaporator under vacuum, to obtain the hydroalcoholic extract of *B. verticillata* (EHBv). EHBv was dissolved in methanol:water (70:30,v/v) for 60 min under mechanical agitation, and successively subjected to liquid-liquid extraction with hexane, chloroform, and ethyl acetate. The solutions were filtered and concentrated at 40°C in a rotary evaporator under vacuum, to ethyl acetate fraction (FAc).

### Phenolic and flavonoid content assessment

Total phenolic content (TPC) was determined using Folin-Ciocalteu reagent and 20% sodium carbonate. The reaction was kept in the dark for 2 h at room temperature; absorbance was read with a spectrophotometer at 760 nm (Dutra et al., 2014). The PCC was calculated based on the calibration curve plotted with gallic acid standard solutions (1.0–30.0 μg/mL) and is expressed as gallic acid equivalent (mg/mL).

Total flavonoid content (TFC) was determined using a 5% methanol solution of aluminum chloride (AlCl_3_). The reaction was kept in the dark for 30 min at room temperature; absorbance was read with a spectrophotometer at 425 nm (Dutra et al., 2008). The TFC was calculated based on the calibration curve plotted with quercetin standard solutions (1.0–30.0 μg/mL) and is expressed as quercetin equivalent (mg/mL).

### High-performance liquid chromatography with ultraviolet-visible detector (HPLC UV/Vis)

EHBv and FAc were analyzed with an HPLC device (Thermo Finnigan Surveyor) coupled to an ultraviolet-visible detector and a reversed phase ACE C-18 (250 X 4.6 mm, 5 μm) column was used. The components of FAc and EHBv were separated at room temperature through gradient elution at a 1 mL/min flow rate. The mobile phases consisted of purified water with 0.1% acetic acid (A) and acetonitrile (B). The gradient used was as follows: 0–5 min, 20% B; 5–10 min, 25% B; 10–15 min, 25–23% B; 15–20 min, 23–21% B; 20–25 min, 21–18% B; 25–30 min, 18–15% B; 30–35 min, 15–0% B. The injection volume was 5 μL, and UV-Vis detection was performed at 254 nm. The compounds were identified on the basis of co-injection with standards.

### Gas chromatography—mass spectrometry (GC-MS)

FAc (10 mg) was derivatized in pyridine (300 μL) and bis-(trimethylsilyl) trifluoroacetamide with trimethylchlorosilane (BSTFA/TMCS, 100 μL) and was heated at 80°C for 1 h. The derivatized product was analyzed with a gas chromatograph (GC-2010, Shimadzu, Japan) coupled to a mass spectrometer (GCMS-QP2010 SE, Shimadzu, Japan) with an Rtx-5MS column (30 m × 0.25 mm ix 0.25 μm, Restek, USA), helium as the carrier gas and a 1.0 mL/min flow rate. The oven temperature was first kept at 70°C and then set to increase 4°C/min until 310°C. The temperature was maintained at 310°C for 4 min. The injector temperature was set to 250°C; the injection volume was 1.0 mL at a 1:30 ratio. The mass spectra were obtained by means of electron impact ionization (70 eV) on total ion scanning mode (40 to 1,000 m/z) with the ion source at 200°C. The compounds were identified through comparison of the obtained mass spectra with the NIST 11 library.

### *In vivo* biological studies

#### Animals

The present study used adult, male and female Wistar *Rattus norvegicus* rats with weights ranging from 200 to 300 g and adult, male, and female *Mus musculus* mice with weights ranging from 25 to 35 g, which were procured from the Central Vivarium (Biotério Central), Federal University of Maranhão (UFMA). Animals were provided with free access to food and water in an environment with controlled temperature and 12/12 h light/dark cycle. This study was carried out in accordance with the recommendations of IASP Guidelines for the Use of Animals in Research. The experimental protocols were approved by the UFMA Ethics in Animal Use Committee (CEUA), ruling no. 17, protocol no. 23115.013545/2015-89.

#### Experimental groups

Six experimental groups with 6 animals each were used. CTRL group was treated oral (p.o) with 0.9% NaCl (0.1 mL/kg); the INDO group was treated with indomethacin (10 mg/kg p.o.); MEM group was treated with memantine (10 mg/kg p.o.); FAc groups were treated with fraction the *B. verticillata* at doses of 25 mg/kg p.o (FAc 25) and 50 mg/kg p.o (FAc 50) and the EHBv group was treated with the hydroalcoholic extract of *B. verticillata* (500 mg/kg p.o.). NaCl 0.9% was used as the vehicle to dissolve the solutions.

After the results obtained in the *in silico* studies, it was observed that of the active compounds ursolic acid (UA) present in the FAc presented better results. Then 6 animals were treated orally with UA (10 mg/kg p.o) and submitted to the carrageenan-induced paw edema test and tail flick.

#### Carrageenan-induced paw edema

This test was performed to assess the pharmacological activities of the investigated compounds after subplantar injection of carrageenan. Mice were distributed and treated as described in the “Experimental groups” section. Sixty minutes after the onset of treatments, paw edema was induced through administration of 50 μL of 1% carrageenan via subplantar injection in the right paw; the same volume of 0.9% NaCl was injected in the left paw. The paw volume was measured with a digital plethysmometer 1, 2, 3, 4, and 5 h after induction. Edema was calculated as the difference between the right and left paw volume and is expressed as paw volume variation (ml) over time (Winter et al., 1962; Sadeghi et al., 2011).

#### Writhing test

The acetic acid-induced writhing test is described as a visceral-somatic inflammatory model used for pharmacological screening of central and peripheral antinociceptive activity. Mice were distributed and treated as described in the “Experimental groups” section. Sixty minutes after treatment onset, abdominal writhing was induced through intraperitoneal administration of 0.8% acetic acid (10 mL/kg). The number of contractions was cumulatively counted for 20 min after induction (Koster et al., 1959). The results are expressed as the average number of cumulative abdominal contractions (Shamsi and Keyhanfar, 2013; Mansouri et al., 2014).

#### Formalin test

The formalin test for nociception allows assessment of the neurogenic nociceptive mechanisms triggered by activation of nociceptive fibers and the inflammatory mechanisms activated following the release of inflammatory mediators. Mice were treated as described above; 60 min later, a subplantar injection of 20 μL of 2.5% formalin was administered in the right paw. The nociceptive response, characterized by paw licking or biting, was observed during the first 5 min to assess neurogenic mechanisms and then from minutes 15 to 30 to assess inflammatory mechanisms (Hunskaar and Hole, 1987; Nemoto et al., 2015).

#### Tail flick test

This test was performed to assess central antinociceptive activity through the stimulation of spinal reflexes. Rats were treated as described above; 60 min later, a thermal stimulus was applied to the final third of the tail (Ugo Basile, Varese-Italy), and the latency to tail flick was measured at baseline, 30, 60, 120, and 180 min. The stimulus intensity was set to obtain 3–6 s latency times; the cutoff point was set to 10 s to avoid tail injury (D'Amour and Smith, 1941; Mansouri et al., 2014).

### *In silico* studies

#### Structure of compounds and receptors

The compounds identified in FAc were obtained from the PubChem Project database and were structurally plotted in 3 dimensions (3D) using GaussView 5.0.8 (Dennington et al., 2009). Geometric and vibrational properties were calculated (optimized) under vacuum by means of the density functional theory (DFT) method using functional hybrid B3LYP combined with basis 6–31 ++ G (d, p) in the Gaussian 09 program (Frisch et al., 2009).

The 3D structure of Swiss mouse COX-2 (chain A) was obtained from the Protein Data Bank (PDB, #1DDX). The 3D structures of the drugs MEM and INDO were obtained from the PubChem Project (CID 4054 and 3715, respectively). The structural model of the *Rattus norvegicus* NMDA receptor subunits GluN1a and GluN2B was obtained by means of homology modeling.

#### Homology modeling

Homology modeling was performed following Ramos et al. (2012) with MODELER 9v14 (Webb and Sali, 2014) and the amino acid sequences of subunits GluN1a and GluN2B (NCBI GI 645985944 and GI 645985945, respectively). As the crystallographic structure of the PDB NMDA (code 4PE5) are not complete, models were generated by homology modeling (HM-GluN1a and HM-GluN2B) using the crystallographic structure of PDB code 4TLL (GluN1/GluN2B NMDA) as template. The quality of the selected models was checked with the programs ProCheck (Laskowski et al., 1993) and Errat (Colovos and Yeates, 1993), run in the SAVES server with Z-Score (ProSA-web Protein Structure Analysis) (Table S2).

#### Molecular docking

The AutoDock 4.2 package (Morris et al., 2008) was used to prepare proteins (refined models) and ligands for docking calculations using the AutoDock Tools (ADT) module, version 1.5.6, according to Ramos et al. (2012). The affinity grid centers were defined on residue Arg120 for COX-2, Tyr513 for NMDA GluN1A and Arg487 for NMDA GluN2B. The initial complex coordinates for MD simulations were selected based on the lowest energy configuration of clusters combined with visual inspection.

#### Molecular dynamics of complexes

The MD simulations of the complexes selected after molecular docking were performed using GROMACS 4.6.7 software (Berk et al., 2014) following Ramos et al. (2012). The ligand topologies were generated with *Automated Topology Builder* (ATB) *and Repository* version 2.1 (Malde et al., 2011). The protonation states of histidine residues were determined using the H++ online server—http://biophysics.cs.vt.edu/hppdetails.php. To enlarge the sample, 3 10-ns MD simulations were performed per complex using different atomic velocities assigned according to the Maxwell distribution. The data generated for the last 4 ns in each simulated system were used for analysis. During the production step, 123 frames were obtained at 100-ps intervals. The details of the interactions were calculated with *LigPlot*++ software (Laskowski et al., 1993). A minimum of 50% of contact (total of hydrophobic interactions and hydrogen bonds) in the analyzed frames was defined as a criterion of binding efficacy.

#### Statistical analysis

Mean values among experimental groups were compared through univariate analysis of variance (one-way ANOVA) followed by the Newman-Keuls test at *p* < 0.05. The data were analyzed in the terms of means ± standard errors using GraphPad Prism 5 software.

## Results

### Phenolic and flavonoid content assessment

The concentrations of total phenolic compounds and flavonoids in EHBv were 9.61 and 7.67 mg/mL, respectively.

### HPLC UV/Vis analysis

Gallic acid, ß-sitosterol, glycyrrhetinic acid, ß-amyrin, caffeic acid, coumaric acid, and quercetin were identified in EHBv (Table 1 and Figure S1). Gallic acid, ursolic acid, caffeic acid, and ellagic acid were identified in FAc (Table 1 and Figure S2).

**Table 1:** **High-performance liquid chromatography (HPLC-UV) analysis of the hydroalcoholic extract (EHBv) and ethyl acetate fraction (FAc) from ***B. verticillata*****.

	**Compounds identified**	**Retention time (Rt) minute**	**Area (%)**
EHBv	Gallic acid	9.6	1.1
	β-sitosterol	12.9	0.1
	Glycyrrhetinic acid	13.9	1.9
	β- amyrin	14.2	26.6
	Caffeic acid	16.4	0.1
	Coumaric Acid	20.0	1.2
	Quercetin	31.7	14.40
FAc	Gallic acid	4.7	7.3
	Ursolic acid	6.3	2.3
	Caffeic acid	6.71	2.3
	Ellagic acid	8.20	6.1

### GC-MS analysis

Thirteen components were detected in FAc, corresponding to alcohols, sugars, fatty acids, and flavonoids. The main components found were the sugars D-psicofuranose (10.2%) and glucopyranose (33.47%) and the fatty acid 10-undecenoic acid (15.70%) (Table 2).

**Table 2:** **Gas chromatography-mass spectrometry (GC-MS) analysis of the ethyl acetate fraction (FAc) from ***B. verticillata*****.

**Class Component**	**Compounds identified**	**Area (%)**
Alcohol	Glycerol	9.7
	Butanetriol	3.0
Sugars	D-galactose	1.5
	Trehalose	1.8
	Fructopyranose	2.4
	D-psicofuranose	10.2
	Glucopyranose	33.4
Fatty acids	Palmitic acid	2.0
	Stearic acid	2.7
	1-monopalmitin	2.2
	Acid 10-undecenoic	15.7
Flavonoids	Quercetin	1.9
	Myricetin	2.1

### *In vivo* biological studies

#### Carrageenan-induced paw edema test

Subplantar injection of carrageenan induced edema on the animals' paws that lasted the full period of observation, i.e., 5 h, with the peak of edema starting 4 h after induction.

**Figure 1:**
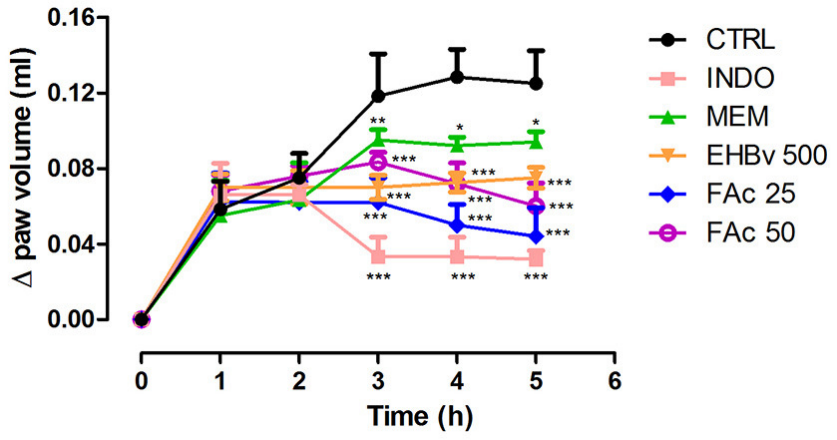
**Paw edema induced by subplantar administration of 1% carrageenan in mice treated orally with NaCl 0.9%, indomethacin 10 mg/kg, memantine 10 mg/Kg, EHBv 500 mg/Kg and FAc (25 and 50 mg/Kg)**. ^*^p < 0.05; ^**^p < 0.01; ^***^p < 0.001 vs. CTRL (ANOVA; Newman Keuls).

Treatments consisting of EHBv 500 and FAc (FAc 25 and FAc 50) significantly reduced carrageenan-induced edema at 3, 4, and 5 h after induction compared to control. The percentage of reduction in edema caused by the treatment was 41, 42, and 43% for EHBv 500; 48, 61, and 67% for FAc 25; and 41, 53, and 61% for FAc 50 respectively. Treatment with INDO significantly reduced edema by 72, 74, and 77%, while MEM reduced edema by 20, 18, and 14% at 3, 4, and 5 h after induction, respectively, compared to control. Comparisons between the effects of *B. verticillata* extract, fractions and standard drugs (indomethacin and memantine) revealed that INDO, FAc 25, and FAc 50 were the most efficacious in reducing edema. The effects of FAc at doses of 25 and 50 mg/kg were equivalent, without statistically significant difference (Figure 1 and Table S1).

#### Writhing test

**Figure 2:**
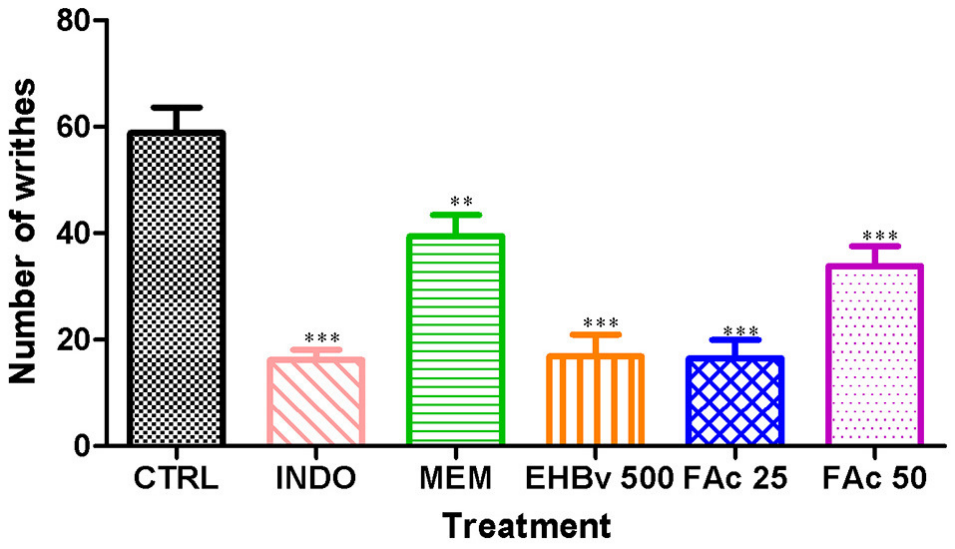
**Writhing induced by the intraperitoneal administration of 0.8% acetic acid (10 ml/kg) in mice treated orally with NaCl 0.9%, indomethacin 10 mg/kg, memantine 10 mg/Kg, EHBv 500 mg/Kg and FAc (25 mg/kg and 50 mg/Kg)**. ^**^p < 0.01; ^***^p < 0.001 vs. CTRL (ANOVA; Newman Keuls).

Intraperitoneal administration of acetic acid caused pain to animals during entire 20 min of assessment. Treatments with EHBv 500, FAc 25, and FAc 50 significantly reduced the number of abdominal contractions by 71, 72, and 42%, respectively, compared to control (Figure 2). Treatment INDO and MEM significantly reduced the number of abdominal contractions by 72.5 and 33%, respectively, compared to control. The effects of INDO, EHBv 500 and FAc 25 on writhing test, and the reductions in abdominal contractions induced by FAc 50 and MEM were equivalent.

#### Formalin test

**Figure 3:**
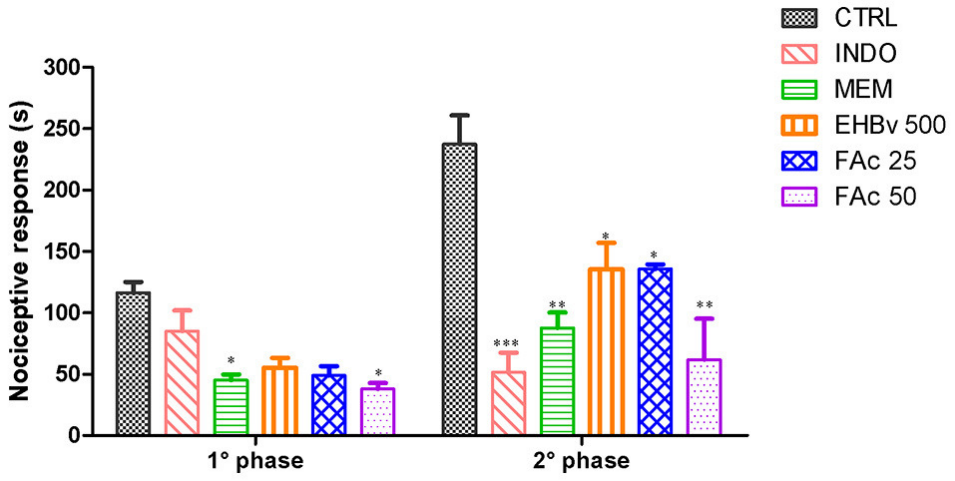
**Formalin test induced by subplantar formalin administration of 2.5% in mice treated orally with NaCl 0.9%, indomethacin 10 mg/kg, memantine 10 mg/Kg, EHBv 500 mg/Kg and FAc (25 and 50 mg/Kg)**. ^*^p < 0.05; ^**^p < 0.01; ^***^p < 0.001 vs. CTRL (ANOVA; Newman Keuls).

This test assessed pain at 2 different stages. In stage 1 MEM, FAc 25, and FAc 50 treatments significantly reduced neurogenic pain by 54, 45, and 57%, respectively, compared to control, while INDO did not induce a significant reduction of neurogenic pain. The effects of FAc 50 and MEM were similar. In stage 2, INDO, MEM, EHBv 500, FAc 25, and FAc 50 treatments significantly reduced inflammatory pain by 82, 63, 57, 57, and 59%, respectively, compared to control (Figure 3). The effects of EHBv 500, FAc 25 and FAc 50 were similar to MEM and INDO treatments, without statistically significant differences.

#### Tail flick test

**Figure 4:**
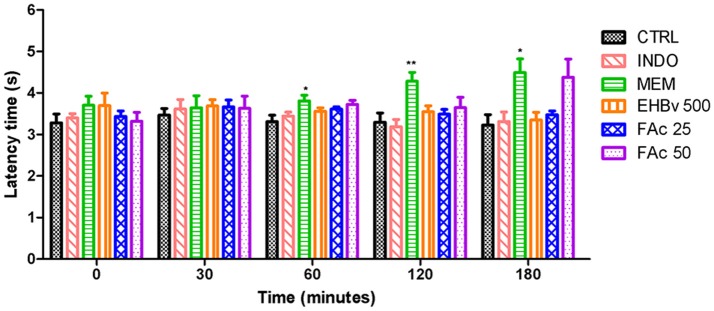
**Tail flick test in rats treated orally with NaCl 0.9%, indomethacin 10 mg/kg, memantine 10 mg/Kg, EHBv 500 mg/Kg and FAc (25 mg/kg and 50 mg/Kg)**. ^*^p < 0.05; ^**^p < 0.01 vs. CTRL (ANOVA; Newman Keuls).

Treatment with MEM significantly reduced pain, as evidenced by 15, 40, and 40% increases in the latency time at 60, 120, and 180 min, respectively, compared to control. INDO, EHBv 500, FAc 25, and FAc 50 were unable to increase the latency time of the animals during the 180 min assessment period (Figure 4).

### *In silico* studies

#### Molecular docking

All compounds identified in FAc on HPLC UV-Vis analysis were selected for molecular docking. The parameters affinity (binding energy [ΔG_bind_] and inhibition constant [Ki]) and values of all ligands are described in Table 3.

**Table 3:** **Interactions by molecular docking of indomethacin, memantine and compounds identified in FAc with COX-2, GluN1a and GluN2B**.

**GluN1a**			**GluN2B**			**COX-2**		
**Ligand**	ΔG_bind_^*^ (kcal/mol)	Ki^**^ (μM)	**Ligand**	ΔG_bind_^*^ (kcal/mol)	Ki ^**^ (μM)	**Ligand**	ΔG_bind_^*^ (kcal/mol)	Ki^**^ (μM)
Memantine	−7.82	1.86	Memantine	−5.66	71.18	Indomethacin	−8.30	0.82
Ursolic acid	−7.02	7.13	Ursolic acid	−5.69	67.35	Ursolic acid	−9.86	0.05
Ellagic acid	−5.67	70.17	Ellagic acid	−5.34	122.58	Ellagic acid	−7.54	2.99
Gallic acid	−4.57	448.26	Gallic acid	−5.16	164.28	Gallic acid	−6.68	12.69
Caffei acid	−4.47	531.44	Caffei acid	−5.04	201.80	Caffei acid	−5.88	49.19

* *ΔG_bind_, binding energy*.

** *Ki, inhibition constant*.

In relation to COX-2, UA exhibited higher ΔG_bind_ values and inhibition constants compared with INDO, which were −9.86 kcal/mol and 0.05 μM and −8.30 kcal/mol and 0.82 μM, respectively. The ΔG_bind_ values corresponding to ellagic acid, caffeic acid, and gallic acid were −7.54, −6.68, and −5.88 kcal/mol, respectively. Relative to the NMDA receptor, UA exhibited the best affinity parameters with GluN1a, with values of −7.02 kcal/mol and 7.13 μM, close to the values obtained for MEM, which were −7.82 kcal/mol and 1.86 μM. With respect to GluN2B, the affinity values of UA were slightly higher compared with MEM, which were −5.69 kcal/mol and 67.35 μM vs. −5.66 kcal/mol and 71.18 μM, respectively. Relative to GluN1a and GluN2B, the ΔG_bind_ values corresponding to ellagic acid, caffeic acid and gallic acid were −5.67, −4.57, and −4.47 kcal/mol and −5.34, −5.16, and −5.04 kcal/mol, respectively.

The interactions of UA, MEM, and INDO with amino acids identified in selected configurations obtained through molecular docking calculations are described in Table 4.

**Table 4:** **Interactions of ursolic acid, memantine and indomethacin for the conformations chosen by molecular docking**.

**GluN1a**			**GluN2B**		**COX-2**	
**Ligand**	**Hydrogen bonds**	**Hydrophobic interactions**	**Hydrogen bonds**	**Hydrophobic interactions**	**Hydrogen bonds**	**Hydrophobic interactions**
Ursolic acid	Tyr535, Arg733	Ile497, Phe507, Ser508, Lys509, Pro510, Ser734, Gly735	Phe493, Lys736	Ile483, Ser420, Glu489, Val491, Asp492	Lys83, Tyr122, Ser471	Pro84, Tyr115, Ser119, Arg120, Leu123, Phe470, Glu524, Pro528
Memantine	Asp765, Asp767	Lys512, Ala712, Glu715, Phe716, Glu764	Val490	Val169, Trp171, Glu489, Lys736, Asp737	NA	NA
Indomethacin	NA	NA	NA	NA	Lys83, Tyr122	Asn43, Arg44, Thr62, Leu80, Lys468, Arg469, Ser471.

*NA, Not rated*.

#### Molecular dynamics simulations

**Figure 5:**
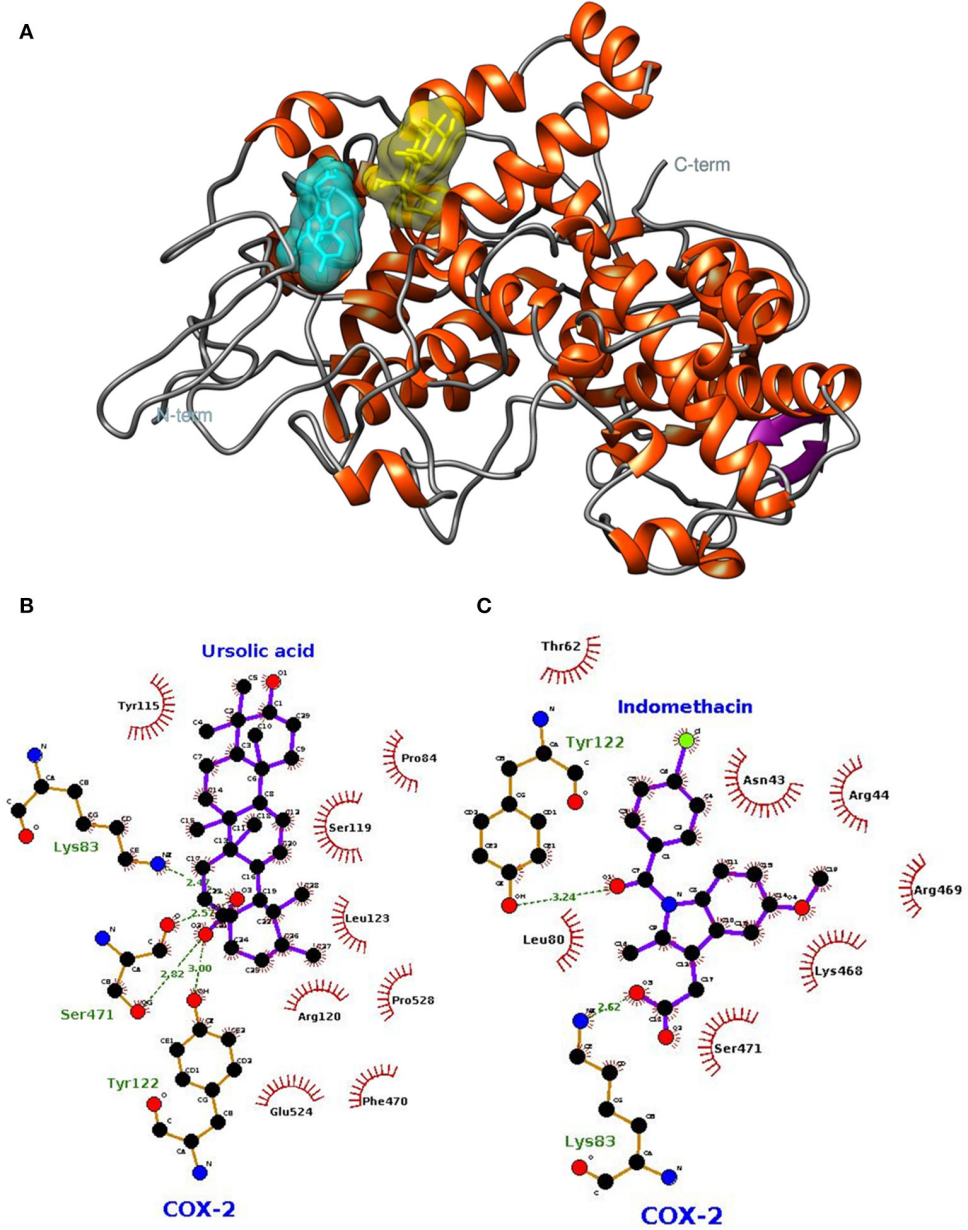
**Spatial conformation obtained by molecular docking of ursolic acid (yellow) and indomethacin (blue) with the COX-2 enzyme (PDB: 1DDX) (A)**. LIGPLOT diagrams for ursolic acid **(B)** and indomethacin **(C)** interaction in COX-2.

**Figure 6:**
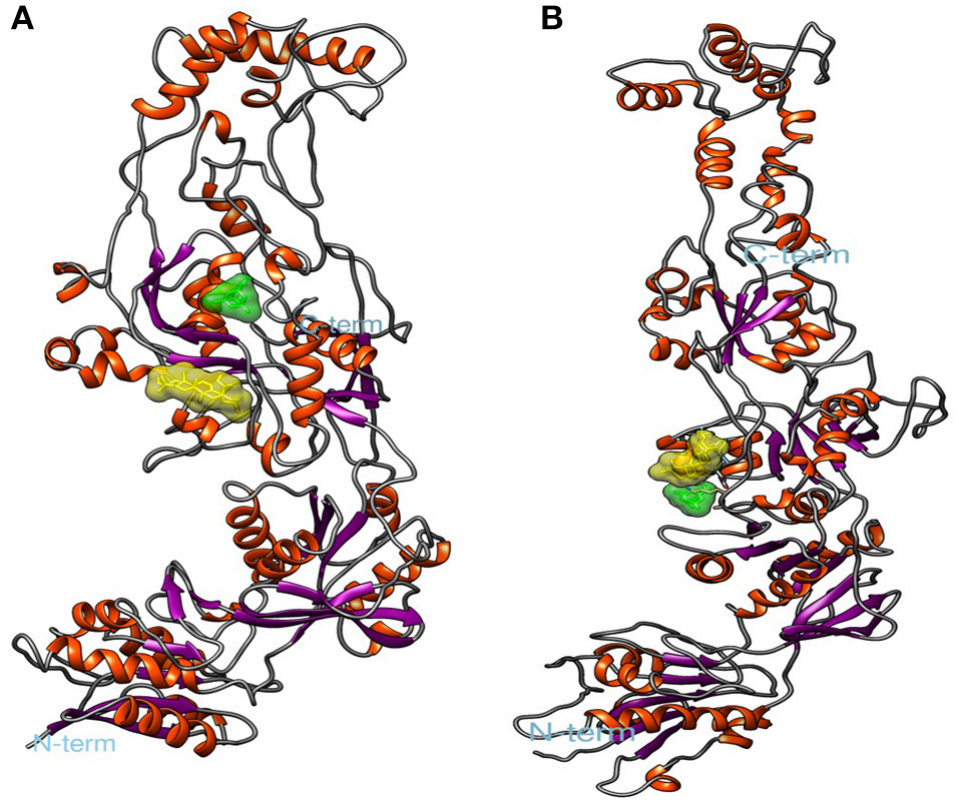
**Spatial conformation obtained by molecular docking of ursolic acid ligands (yellow) and memantine (green) with N-methyl-D-aspartate (NMDA). (A)** GluN1a, **(B)** GluN2B. Image generated by VMD.

The lowest docking-energy conformation of the cluster with lowest energy was chosen as initial structure for the molecular dynamics simulations of the COX-2 (Figures 5A–C) GluN1a (Figure 6A and Figure S3) and GluN2B (Figure 6B and Figure S4) (UA, INDO or MEM) complexes. Interactions ≥50% were considered to be relevant.

**Figure 7:**
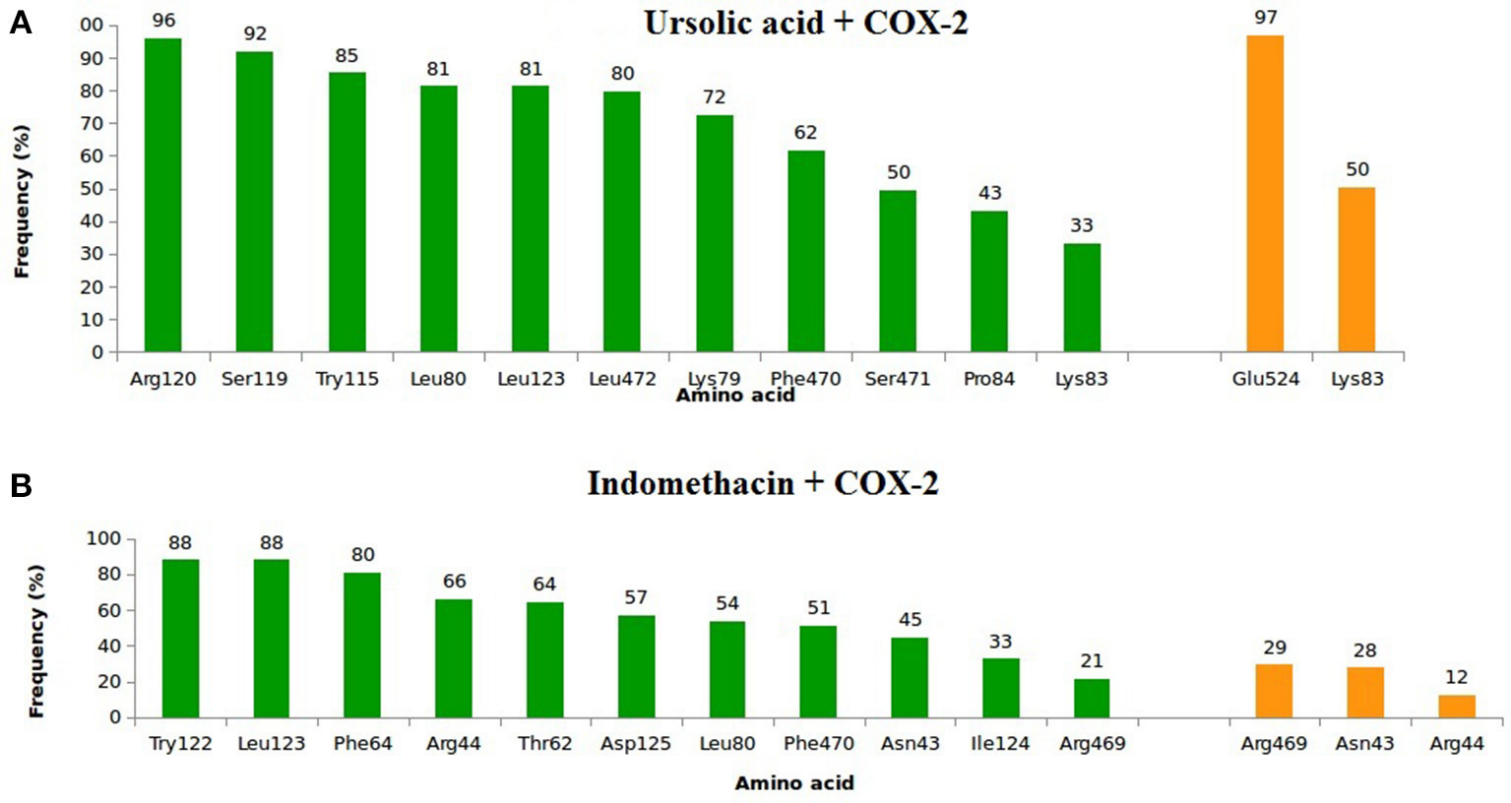
**Frequency of hydrophobic contacts (green) and hydrogen bonding (orange) of ursolic acid + COX-2 (A) and indomethacin + COX-2 (B)**. The frequencies correspond to the end 4 of molecular dynamics simulations.

In relation to COX-2, UA exhibited high frequencies of interaction with Lys79, Leu80, Lys83, Pro84, Arg120, Leu123, Trp155, Ser199, Phe470, Leu472, Ser481, and Glu524 (Figure 7A). The highest frequencies of interaction corresponded to Glu524, with 97% of hydrogen bonds, and Arg120, with 96% of hydrophobic interactions. INDO exhibited high frequencies of interaction with Asn43, Arg44, Thr62, Phe64, Leu80, Trp122, Leu123, lle124, Asp125, Phe470, and Arg469; the most relevant interactions corresponded to Trp122 and Leu123, both with 88% of hydrophobic interactions (Figure 7B).

**Figure 8:**
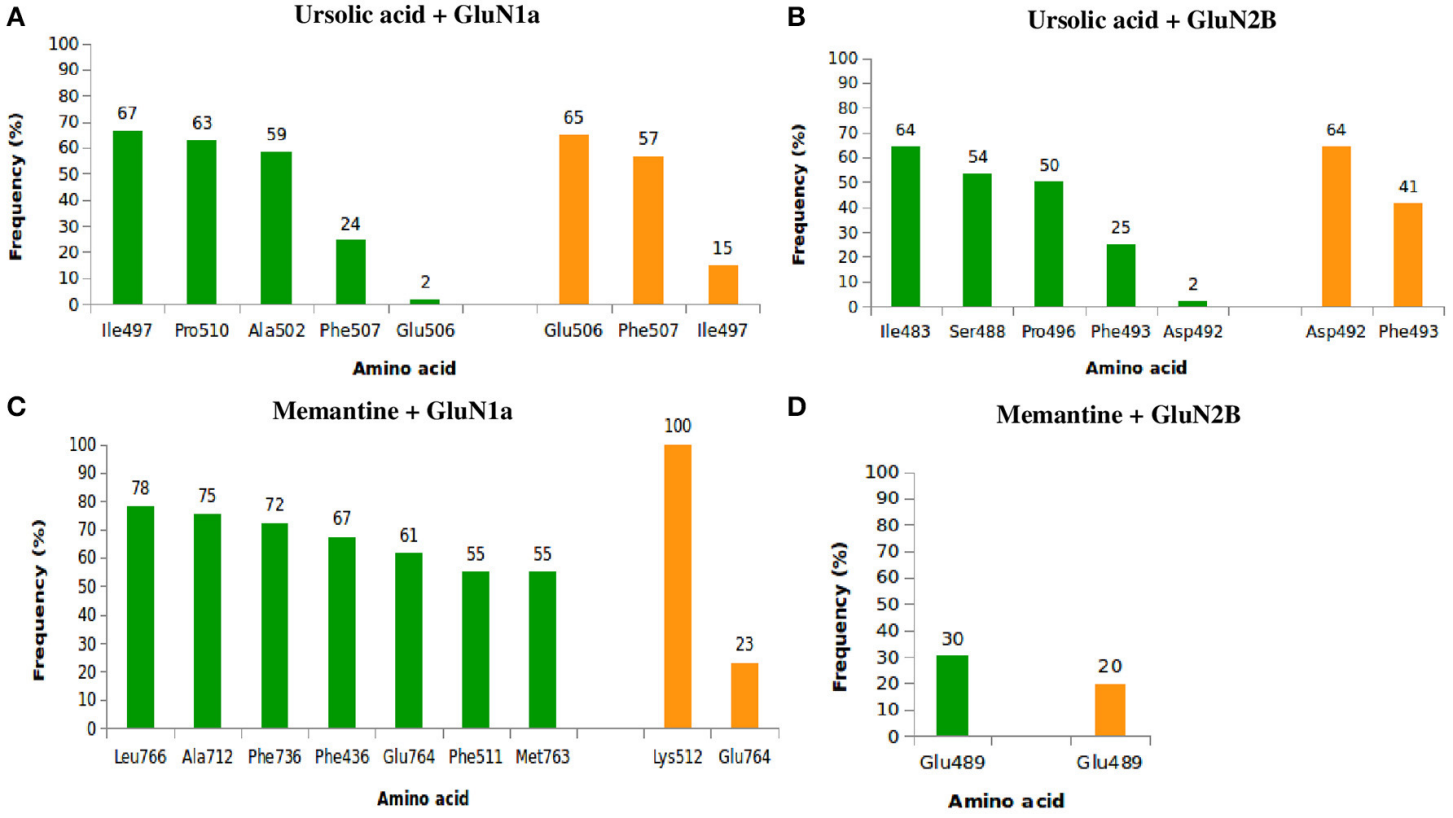
**Frequency of hydrophobic contacts (green) and hydrogen bonding (orange) between ursolic acid + GluN1a (A)**, ursolic acid + GluN2B **(B)**, memantine + GluN1a **(C)**, and memantine + GluN2B **(D)**.

With respect to (Figure 8A), UA exhibited high frequencies of interaction with lle497, Pro510, Ala502, Phe507, and Glu506, with the highest frequencies being 82% with Ile497 (67% of hydrophobic interactions and 15% of hydrogen bonds) and 81% with Phe507 (24% of hydrophobic bonds and 57% of hydrogen bonds). Relative to GluN2B (Figure 8B), UA exhibited high frequencies of interaction with lle483, Ser488, Phe493, Asp492, and Pro496. The frequencies of interaction with Asp492 and Phe493 were both 66%, corresponding to 2 and 25% of hydrophobic bonds and 64 and 41% of hydrogen bonds, respectively. MEM had maximum contact with GluN1a (Figure 8C) and high frequencies of interaction with Phe436, Phe511, Lys512, Ala712, Phe736, Glu764, Met763, and Leu766, with 100% of hydrogen bonds with Lys512 and 84% of interactions with Glu764 (61% of hydrophobic interactions and 23% of hydrogen bonds). Regarding to GluN2B (Figure 8D), MEM exhibited 50% interaction with Glu489 (30% of hydrophobic interactions and 20% of hydrogen bonds).

The frequencies of interaction of UA and MEM with GluN1a and GluN2B are described in Figure 8.

#### Test *in vivo* of *in silico* selected compound

After analysis of the results obtained from the *in silico* tests, tests of Carrageenan-induced paw edema and Tail flick with UA (Sigma-Alderich) were performed.

**Figure 9:**
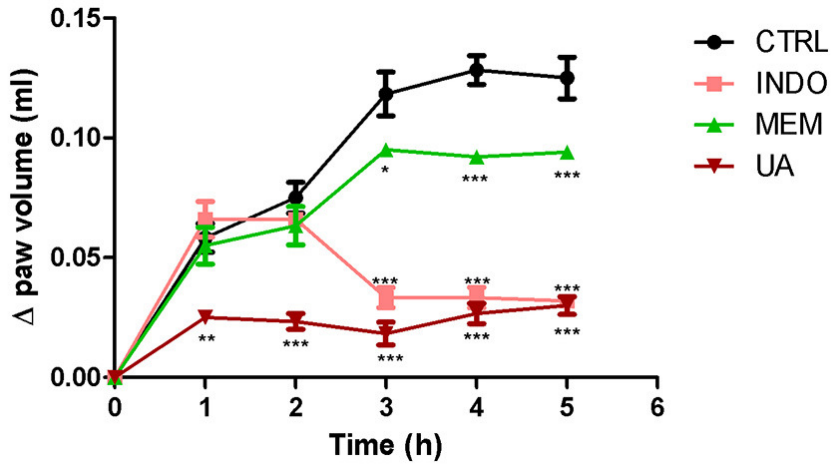
**Paw edema induced by subplantar administration of 1% carrageenan in mice treated orally with NaCl 0.9%, indomethacin 10 mg/kg, memantine 10 mg/Kg, and ursolic acid 10 mg/Kg**. ^*^p < 0.05; ^**^p < 0.01; ^***^p < 0.001 vs. CTRL (ANOVA; Newman Keuls).

Treatment with UA (10 mg/kg) significantly reduced carrageenan-induced edema by at 5 h analyzed. These reductions were 57–84% when compared to the control group. Comparisons of the effects UA, INDO, and MEM revealed that UA were most efficacious in reducing edema. Treatment with INDO and of MEM reduced significantly the paw edema in the 3, 4, and 5 h when compared with CTRL. These reductions were similar to those mentioned above (Figure 9).

**Figure 10:**
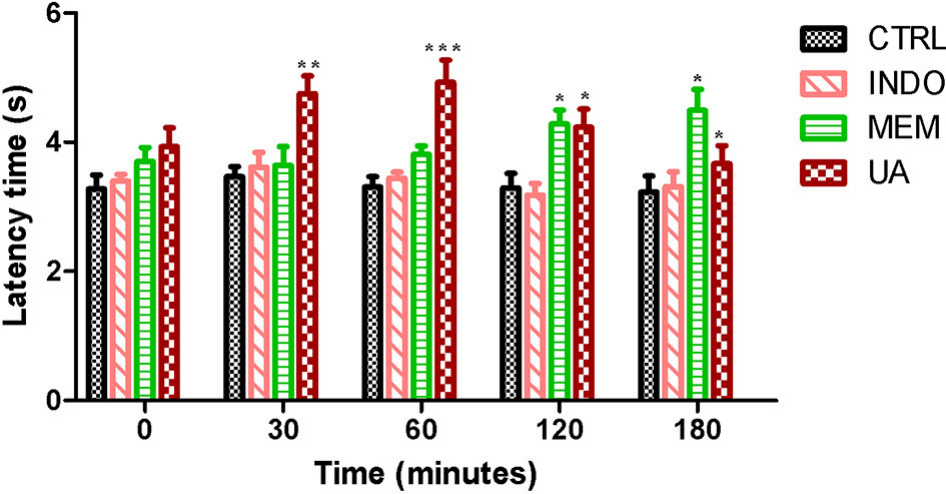
**Tail flick test in rats treated orally with NaCl 0.9%, indomethacin 10 mg/kg, memantine 10 mg/Kg, and ursolic acid 10 mg/Kg**. ^*^p < 0.05; ^**^p < 0.01; ^***^p < 0.001 vs. CTRL (ANOVA; Newman Keuls).

In tail flick test, treatment with UA (10 mg/kg) significantly increased the animal latency time at 30–120 min at 28–42% when compared to control. This effect was not observed after 180 min. Treatment with MEM significantly increased latency time when compared to control. These results were similar to those mentioned above (Figure 10).

## Discussion

The assays performed in the present study showed that *B. verticillata* had peripheral antinociceptive effects and anti-inflammatory properties. The *in silico* tests indicated that UA was chief among the active components identified in FAc, exhibiting relevant interactions with amino-acid residues in COX-2 and NMDA receptor active sites.

The FAc doses (25 and 50 mg/kg) used in this study were chosen according to results obtained in previous studies developed by our research group. Our preliminary studies with EHBv demonstrated that doses 250 and 500 mg/kg had an antinociceptive effect. In order to define the doses used in FAc, we reduced the EHBv dose by 100 times, resulting in doses of 25 and 50 mg/kg.

The anti-edematous effects of EHBv 500, FAc 25, and FAc 50 were similar to INDO starting 3 h after induction. Studies have shown that the paw edema test has a maximum parameter in 3–4 h lasting up to 5 h. After this period of time, induction loses its effectiveness and due to this reason we do not continue the evaluation (Eisenberg et al., 1994; Castardo et al., 2008). Paw edema characteristically develops in 2 stages: the first stage begins with carrageenan administration and lasts up to 2 h, corresponding to the exudative phase of inflammation, which is triggered by the release of inflammatory mediators such as histamine, serotonin and bradykinin. The second stage begins 3 h after carrageenan administration and is characterized by neutrophil infiltration and is sustained by prostanoids (Wilches et al., 2015; Honmore et al., 2016). Along the inflammatory process, or when tissue damage occurs, several substances are released that promote vasodilation, plasma extravasation and cell recruitment. In addition, glutamate release by primary afferent fibers (class Aδ and C) is increased, as is the activation of glutamatergic receptors, such as NMDA, with consequent propagation of action potentials and the release of excitatory neurotransmitters in the posterior horn of the spinal cord (Miller et al., 2011). Accordingly, the anti-edematous effect of *B. verticillata* FAc might be attributed to COX-2 inhibition and the consequent reduction of PGE_2_ synthesis, as it also occurs in the case of oral treatment with INDO. In addition, previous studies showed that blockade of the NMDA receptor also influences inflammation, as it reduces leukocyte migration (Bong et al., 1996) proinflammatory cytokine induction (Morel et al., 2013) and partially inhibits enzyme phospholipase A_2_ (Buritova et al., 1996); all of which explain the anti-edematous effect of MEM.

The EHBv 500, FAc 25, and FAc 50 treatments reduced the number of abdominal contractions on the writhing test by 71, 72, and 42%, respectively. The effects of EHBv 500 and FAc 25 were equivalent to INDO and the effect of FAc 50 was equivalent to MEM.

The writhing test is a visceral inflammatory pain model used for screening compounds with peripheral analgesic activity; it involves activation of somatic and visceral receptors in addition to induction of local inflammation mediated by prostaglandins, bradykinin, tumor necrosis factor (TNF) α and interleukins (ILs) 1β and 8 (Rodrigues et al., 2012; Olonode et al., 2015). Some studies have demonstrated increased activation of peripheral receptors and elevated spinal cord glutamate concentrations in inflammatory pain models (Santos et al., 2005; Shamsi and Keyhanfar, 2013). As the effects of EHBv 500 and FAc 50 treatments were equivalent to INDO, the results of the present study might be mainly attributed to COX-2 inhibition and the consequent reduction of PGE_2_ synthesis. In addition, we must also bear in mind the peripheral inhibition of the action of glutamate on nociceptors induced by MEM, as the effects of this drug and FAc 50 were equivalent.

The results of the present study showed that oral treatment with MEM, FAc 25, and FAc 50 reduced the nociceptive response in both phases of the formalin test. Treatments with EHBv and INDO reduced the nociceptive response time only in the inflammatory phase. The reductions in the nociceptive response induced by *B. verticillata* extract and FAc were similar to MEM and INDO in the first and second phases of formalin test, respectively.

The formalin-induced nociception model involves a biphasic nociceptive response. The first phase (0–5 min) is neurogenic, when class C fibers are predominantly activated; the second phase (15–30 min) corresponds to inflammation and depends on a combination of inflammatory mediators and peripheral and central sensitization (Hunskaar and Hole, 1987; Tjolsen et al., 1992; Liberato et al., 2003).

Peripheral NMDA receptors contribute to the triggering and maintenance of peripheral sensitization under conditions characterized by cell damage and inflammation (Christoph et al., 2005). Several studies have shown that NMDA antagonists, such as MEM, are able to reduce the nociceptive response during both the first and second phases (Davidson and Carlton, 1998; Liberato et al., 2003). McRoberts et al. (2011) found a 50% reduction of the inflammatory phase in GluN1-knockout rats, which suggests that reduced expression of the NMDA receptor decreases glutamate- and substance-P-mediated synaptic signaling in the spinal cord.

NSAIDs such as INDO decrease nociception in the second phase of the nociceptive response only (Tjolsen et al., 1992). On those grounds, one might attribute the reduction of nociception in the first phase to the inhibition of the peripheral action of glutamate on nociceptors and, in the second phase, inhibition of COX-2 and PGE_2_ synthesis.

The tail flick test was performed in the present study to investigate the central analgesic potential of EHBv and FAc. The EHBv 500, FAc 25, FAc 50, and INDO treatments were not able to promote central analgesia. In turn, the latency periods were longer (60, 120, and 180 min after treatment, respectively) than with 10 mg/kg MEM. According to Danneman et al. (1994), the tail behavioral response to nociceptive stimuli is predominantly regulated by spinal and supraspinal structures. Thus, the analgesic effect of MEM is due to the blockade of the NMDA receptor, with consequent reduction of central sensitization mediated by excitatory neurotransmitters, such as glutamate (Parsons et al., 1999; Morel et al., 2013).

The absence of the dose-dependent effect can be explained by reducing the dissolution of the higher dose and the fact that probably the largest dose does not have the same amounts of active compounds present at the lowest dose.

On the basis of *in vivo* tests results, a peripheral antinociceptive activity of *B. verticillata* can be attributed to a reduction in the activity of COX-2 and the peripheral NMDA receptor. In addition the peripheral antinociceptive activity of *B. verticillata* detected in the present study agrees with the findings described by Abdullahi-Gero et al. (2014b). In that study, oral and intraperitoneal treatments with the ethanolic extract of *B. verticillata* leaves in doses of 200–1,000 mg/kg exhibited peripheral and central analgesic and anti-inflammatory effects; furthermore, the results suggested that the 50% lethal dose (LD_50_) for mice and rats is ≥5,000 mg/kg.

According to the chemical analysis results, the biological activity of *B. verticillata* might be mainly attributed to phenolic compounds and triterpenes, since constituents of these metabolite classes were identified in EHBv and FAc. Some studies showed that these compounds have anti-inflammatory and analgesic effects, as follows: gallic acid—reductions in allodynia and anti-inflammatory effects (Angélica et al., 2013); caffeic acid—reductions in leukocyte migration and free radical and nitric oxide production (Mehrotra et al., 2011); ellagic acid—interaction with opioid receptors (Mansouri et al., 2014) and UA—inhibition of nuclear factor-kappa B (NF-kB) activity (Takada et al., 2010).

In order to determine the mechanisms and possible multi-targets of the peripheral antinociceptive activity of *B. verticillata*, the compounds identified in the FAc were submitted to *in silico* studies. Gallic acid, caffeic acid, ellagic acid, and UA were subjected to molecular docking analysis, targeting COX-2, and NMDA receptor.

According to Guimarães et al. (2014), negative ΔG_bind_ values represent favorable interactions of the ligand-receptor complex. The molecular docking interactions in Table 3 demonstrated that UA had lower binding energies with the NMDA receptor subunits GluN1a (−7.02 kcal/mol), GluN2B (−5.69 kcal/mol) and COX-2 (−9.89 kcal/mol). Therefore, the UA was the ligand that presented better interactions with the NMDA receptor and COX-2, hence it was selected for MD simulations and *in vivo* tests.

UA interacted with COX-2 and both NMDA receptor subunits as well as the standard drugs INDO and MEM. From a structural point of view, the COX-2 active site consists of a lipophilic channel with a gate formed by residues Arg120, Tyr355, and Glu524 (Rowlinson et al., 2003), and its activation leads to metabolic changes in arachidonic acid (AA) based on interactions with COX-2 residues Arg120, Tyr355, Tyr385, and Ser530, resulting in prostaglandin production (Xu et al., 2014).

Several studies have confirmed the relevance of interactions involving the aforementioned amino acids, thus pointing to them as potential targets in the investigation of COX-2 inhibitors. Fenamic acid derivatives were assessed for COX-2 inhibitory actions *in vitro*; once the efficacy of the compounds was established, the complexes were subjected to x-ray crystallography. The results indicated interactions of fenamic acid derivatives with Arg120, Tyr355, Tyr385, Trp387, Glu524, and Leu531 (Orlando and Malkowski, 2016). *In vitro* evaluation of the COX-2 inhibitory potential of meloxicam and isoxicam followed by x-ray crystallography detected interactions of both compounds with Met113, Leu117, Arg120, Ile345, Val349, Leu352, Leu359, Phe518, Ala527, Ser530, and Leu531 (Xu et al., 2014). The interaction of COX-2 with ibuprofen, another NSAID with anti-inflammatory and analgesic activities, involved the participation of COX-2 residues Arg120, Val349, Tyr355, Trp387, Met522, Val523, Gly526, Ala527, and Ser530 (Orlando et al., 2015). The results of that study further showed that residues Arg120 and Tyr355, located in the COX-2 channel gate, are crucial for the enzyme's interaction with ibuprofen.

Constant interactions between UA and significant amino acid residues, such as Arg120, Glu524, and others closed related to residues described in previous studies, such as Trp115 (close to Met113 and Leu117, involved in meloxicam and isoxicam interactions), Leu123 (close to Arg120, involved in meloxicam, isoxicam and ibuprofen interactions), and Glu524 (close to Val523, involved in meloxicam and ibuprofen interactions, and to Gly526, involved in ibuprofen interactions).

The present study further found that the UA and COX-2 interaction profile detected upon molecular docking persisted in the MD simulations, even in the presence of temperature, pressure, water, and protein flexibility variations. In addition, the interactions of UA with amino acid residues adjacent to the COX-2 active site and the AA metabolism site, such as Tyr115, Ser119, Tyr122, Leu123, Asp125, and Pro528, might also intensify the COX-2 inhibitory potential of UA. On the basis of the referred facts: (1) UA might block the access of ligands to the COX-2 active site; (2) interactions with Arg120 and amino acid residues adjacent to the COX-2 active site might reduce arachidonic acid metabolization; and (3) interactions of COX-2 active site inhibitors with Arg120 and Glu524 have already been described, which demonstrates the relevance of these amino acid residues for the possible blocking of the COX-2 active site by UA (Figure S5).

From a structural point of view, the NMDA receptor is a heterotetramer formed by 2 subunits, GluN1 and GluN2. These subunits are modulated by domains ATD, LBD, TMD, and CTD (Karakas and Furukawa, 2014). Activation of the NMDA receptor requires simultaneous binding of glutamate to GluN2 and glycine or d-serine to GluN1 through the interactions of the latter α-amino and α-carboxyl groups with LBD regions. The LBD is formed by 3 transmembrane regions (M1, M2, and M3) and 2 segments (S1 and S2), which comprise the active site and are located in subdomains D1 and D2, respectively (Traynelis et al., 2010).

According to Kaye et al. (2006), the active site of subdomain D1 is formed by amino acid residues Gln405, Phe484, Thr486, Pro516, Thr518, and Arg523, and the active site of subdomain D2 is formed by Ser688, Ser687, Val6869, Trp731, Asp732, and Ser756. Several studies with GluN1 antagonists, such as 5,7-dichlorokynurenic acid (DCKA) and 1-thioxo-1,2-dihydro-[1,2,4]triazolo[4,3-a]quinoxalin-4(5H)-one (TK40), verified interactions with Phe484, Thr518, Pro516, and Arg523 (Furukawa and Gouaux, 2003) for DCKA and with Glun405, Phe484, Pro516, Leu517, Thr518, Arg523, Ser687, Ser688, and Val689 for TK40 (Kvist et al., 2013). These interactions correspond to residues Gln383, Phe462, Pro494, Leu495, Thr496, Arg501, Ser665, Ser666, and Val667 in our structure. After 10 ns MD simulations, we found high frequencies of interactions of UA with lle497, Ala502, Phe507, Ser508, Lys509, and Pro510, located close to the sites indicated by previous authors, which points to the potential of UA to inhibit glycine binding to GluN1.

Lee et al. (2014) developed a crystallographic structure of GluN2B complexed with the partial agonist trans-1-aminocyclobuthane-1,3-dicarboxylic acid (t-ACBD). Those authors revelead that t-ACBD interacted with the amino acid residues His479 and Thr507, which are equivalent to His454 and Thr482 in the present study. Addicionally, UA presented interactions with Ile483, Ser48, and all other neighboring amino acid residues, thus demonstrating that UA might prevent binding of agonists to the NMDA receptor GluN2B subunit.

Through *in silico* tests was demonstrated that UA has the COX-2 enzyme and NMDA receptor as multi-targets. The interactions of UA with these two pharmacological targets suggest that there is inhibition of COX-2 activation, NMDA receptor and consequently reduction of the painful process. Compounds and multi-target drugs are currently a promising alternative for the treatment of complex and multifactorial pathologies. They present advantages over polypharmaceutical therapies such as: patient adherence to treatment, improved kinetics and therapeutic efficacy, increased therapeutic range, reduction of drug interactions, adverse and toxicological effects (Talevi, 2015; Nicolik et al., 2016). Because UA has exhibited this feature (multi-targets), it may be considered an advantage over other compounds described in the literature. Verano et al. (2013) has shown that UA 10 mg/kg (i.p) has an antinociceptive effect, so we chose this dose to test its effect orally and have confirmed the antinociceptive and anti-inflammatory effects of UA through inhibitions of the TRPV1 receptor and serotonergic synergism (5-HT); reduced IL-2, IL-6, interferon (IFN)-γ, TNF-α, reactive oxygen species (ROS), phospholipase A2 and NF-κβ release; reduced COX-2 and inducible nitric oxide synthase (iNOS) expression; (Kashyap et al., 2016) and favorable interactions for complexes formed with COX-1 and COX-2 (Magalhães et al., 2012). Zhang et al. (2013) demonstrated that UA can induce apoptosis of cancer cells by reducing COX-2 expression and Ma et al. (2014) demonstrated that UA reduced COX-2 expression in CCl_4_-treated animals. The results presented here evidenced that UA exerts peripheral antinociceptive effect by inhibiting COX-2 in carrageenan- induced paw edema test and analgesic central in the tail flick test.

Until now there are no studies performed *in vivo* or *in silico* to analyse the antinociceptive potential of compounds present in the aerial parts of *B. verticillata*. We suggest that the peripheral antinociceptive effect of this plant species is mainly due to actions of anti-inflammatory components. Possibly contributing to reducing the activity of the enzyme COX-2 and peripheral NMDA receptors, with consequent reductions in pain. *In silico* and *in vivo* studies allowed the selection and suggestion of UA as the main active compound in *B. verticillata*, as it exhibited desirable affinity parameters, stable interactions with COX-2 and NMDA receptor subunits GluN1a and GluN2B and presented peripheral and central analgesic effect. Thus, the UA is configured a promising molecule for the development of COX-2 inhibitors and NMDA receptor antagonists.

## Author contributions

Conceived and designed the experiments: RS, MC, JD. Methodology: RS, NL, AL, CV, FL, JD, LD, RR. Analyzed the data: RS, AL, CV, JD, LD, RR, MC, JG. Contributed reagents/materials/analysis tools: RR, MR. Wrote the paper: RS, AL, CV, JD, LD, RS, MC, JD.

## Funding

The present study was funded by the Maranhão Research Foundation (FAPEMA) and the National Council for Scientific and Technological Development (CNPq).

### Conflict of interest statement

The authors declare that the research was conducted in the absence of any commercial or financial relationships that could be construed as a potential conflict of interest.
