# Supplementary material for: Antinociceptive Activity of Borreria verticillata: In vivo and In silico Studies
Source: Front Pharmacol. 2017 May 22;8:283. doi: 10.3389/fphar.2017.00283 (PMC5439013; doi:10.3389/fphar.2017.00283)
Supplement: Supplementary file 1 [file Table1.doc]

**Table S1. Paw edema induced by subplantar administration of 1% carrageenan in mice treated orally with NaCl 0.9%, indomethacin 10 mg/kg, memantine 10 mg/Kg, EHBv 500 mg/Kg and FAc (25mg/kg and 50 mg/Kg).**

|  | **Tratement** | | | | | |
| --- | --- | --- | --- | --- | --- | --- |
| **Time (h)** | **CTRL** | **INDO** | **MEM** | **EHBv** | **FAc 25** | **FAc 50** |
| **1** | 0.06±0.006 | 0.07±0.007 | 0.05±0.008 | 0.05±0.013 | 0.06±0.004 | 0.07±0.003 |
| **2** | 0,07±0.006 | 0.07±0.002 | 0.06±0.008 | 0.05±0.009 | 0.08±0.004 | 0.07±0.004 |
| **3** | 0.12±0.009 | 0.03±0.004 ^***^ | 0.09±0.002 ^**^ | 0.06±0.005 ^***^ | 0.07±0.004 ^***^ | 0.07±0.002 ^***^ |
| **4** | 0.12±0.006 | 0.03±0.004 ^***^ | 0.10±0.012 ^*^ | 0.05±0.004 ^***^ | 0.06±0.004 ^***^ | 0.07±0.002 ^***^ |
| **5** | 0.14±0.004 | 0.03±0.002 ^***^ | 0.12±0.008 ^*^ | 0.05±0.006 ^***^ | 0.05±0.006 ^***^ | 0.08±0.004 ^***^ |

The values represent the mean ± standard error. * *p<* 0,05; ** *p<* 0,01; ****p<* 0,001 versus CTRL (ANOVA; Newman Keuls).
