# Supplementary material for: Antinociceptive Activity of Borreria verticillata: In vivo and In silico Studies
Source: Front Pharmacol. 2017 May 22;8:283. doi: 10.3389/fphar.2017.00283 (PMC5439013; doi:10.3389/fphar.2017.00283)
Supplement: Supplementary file 2 [file Table2.DOC]

**Table S2. Comparison of quality of templates structures with the homology model**

| Template  or model | Procheck | | | | | Errat | Z-Score |
| --- | --- | --- | --- | --- | --- | --- | --- |
|  | Core^a^ | Allow.^b^ | Gener.^c^ | Disall.^d^ | Overall G-factor^e^ |  |  |
| GluN1 | 85.3% | 13.8% | 0.8% | 0.2% | -0.22 | 93.57 | -10.30 |
| GluN2B | 87.1% | 12.6% | 0.2% | 0.2% | -0.30 | 87.37 | -9.57 |
| HM-GluN1 | 71.6% | 25.1% | 1.6% | 1.6% | -0.73 | 93.06 | -10.39 |
| HM-GluN2B | 68.1% | 28.9% | 1.4% | 1.6% | -0.78 | 89.77 | -8,30 |

^a^ percentage of residues in most favored regions; ^b^ percentage of residues in permitted areas; ^c^ percentage of residues in regions generously allowed; ^d^ percentage of residues in areas not permitted ^e^ _dihedral G-factor._.
