# Supplementary material for: Antinociceptive Activity of Borreria verticillata: In vivo and In silico Studies
Source: Front Pharmacol. 2017 May 22;8:283. doi: 10.3389/fphar.2017.00283 (PMC5439013; doi:10.3389/fphar.2017.00283)
Supplement: Supplementary file 3 [file Image1.PDF]

A

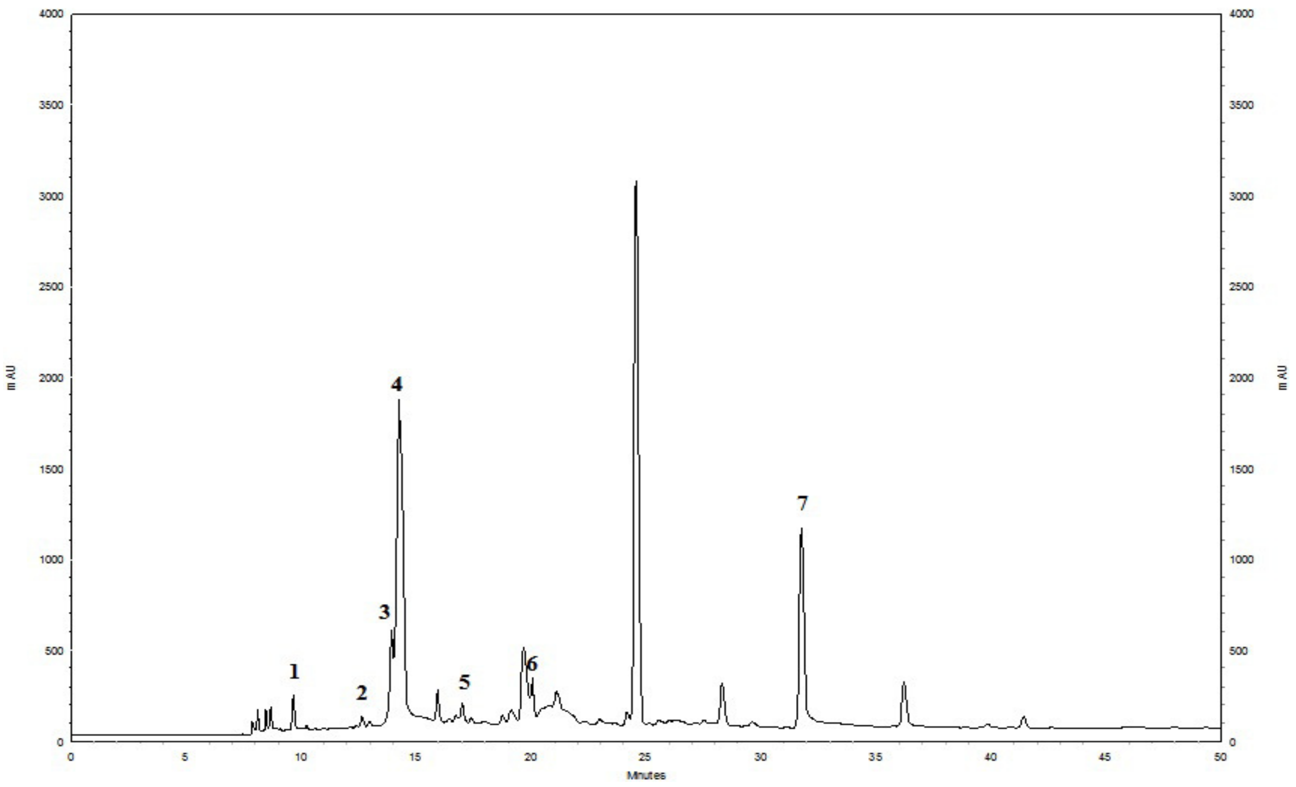

B

Compounds identified in hydroalcoholic extract of *B.verticillata* by HPLC/UV-Vis.

Peak no.	Identification
1	Gallic acid
2	β-sitosterol
3	Glycyrrhetinicacid
4	β-amyrin
5	Caffeic acid
6	Coumaric acid
7	Quercetin

Figure S1. HPLC chromatogram of compounds identified in the hydroalcoholic extract of *Borreria verticillata* (A). Peak numbers follow those listed (B).
