# Supplementary material for: Antinociceptive Activity of Borreria verticillata: In vivo and In silico Studies
Source: Front Pharmacol. 2017 May 22;8:283. doi: 10.3389/fphar.2017.00283 (PMC5439013; doi:10.3389/fphar.2017.00283)
Supplement: Supplementary file 4 [file Image2.PDF]

A

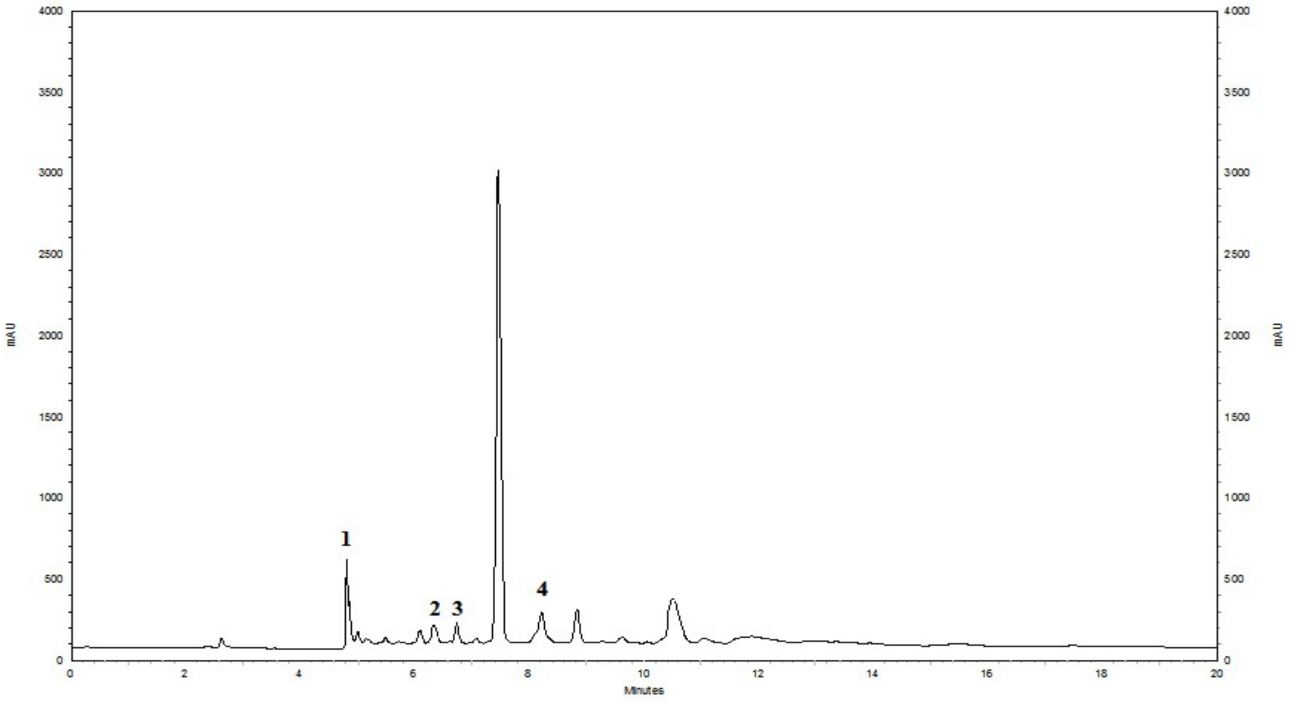

B

Compounds identified in ethyl acetate fraction of *B.verticillata* by HPLC/UV-Vis

Peak no.	Identification
1	Gallic acid
2	Ursolic acid
3	Caffeic acid
4	Ellagic acid

Figure 2. HPLC chromatogram of compounds identified in the ethyl acetate fraction of *Borreria verticillata* (A). Peak numbers follow those listed (B).
