# Supplementary material for: Antinociceptive Activity of Borreria verticillata: In vivo and In silico Studies
Source: Front Pharmacol. 2017 May 22;8:283. doi: 10.3389/fphar.2017.00283 (PMC5439013; doi:10.3389/fphar.2017.00283)
Supplement: Supplementary file 5 [file Image3.PDF]

Supporting Information

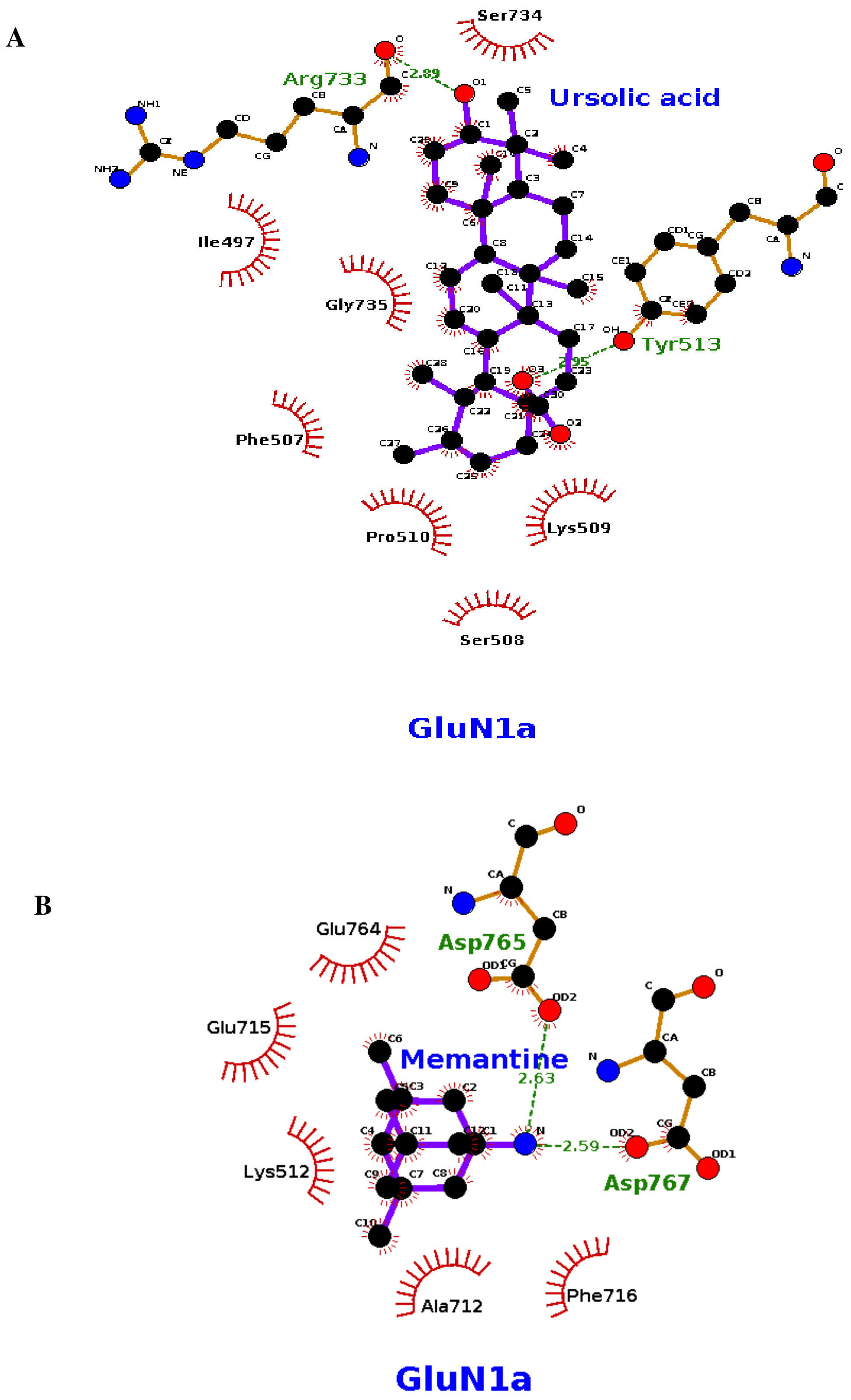

Figure S3. LIGPLOT diagrams for ursolic acid (A) and memantine (B) interaction in GluN1a.
