# Supplementary figures and images for: Antinociceptive Activity of Borreria verticillata: In vivo and In silico Studies
Source: Front Pharmacol. 2017 May 22;8:283. doi: 10.3389/fphar.2017.00283 (PMC5439013; doi:10.3389/fphar.2017.00283)

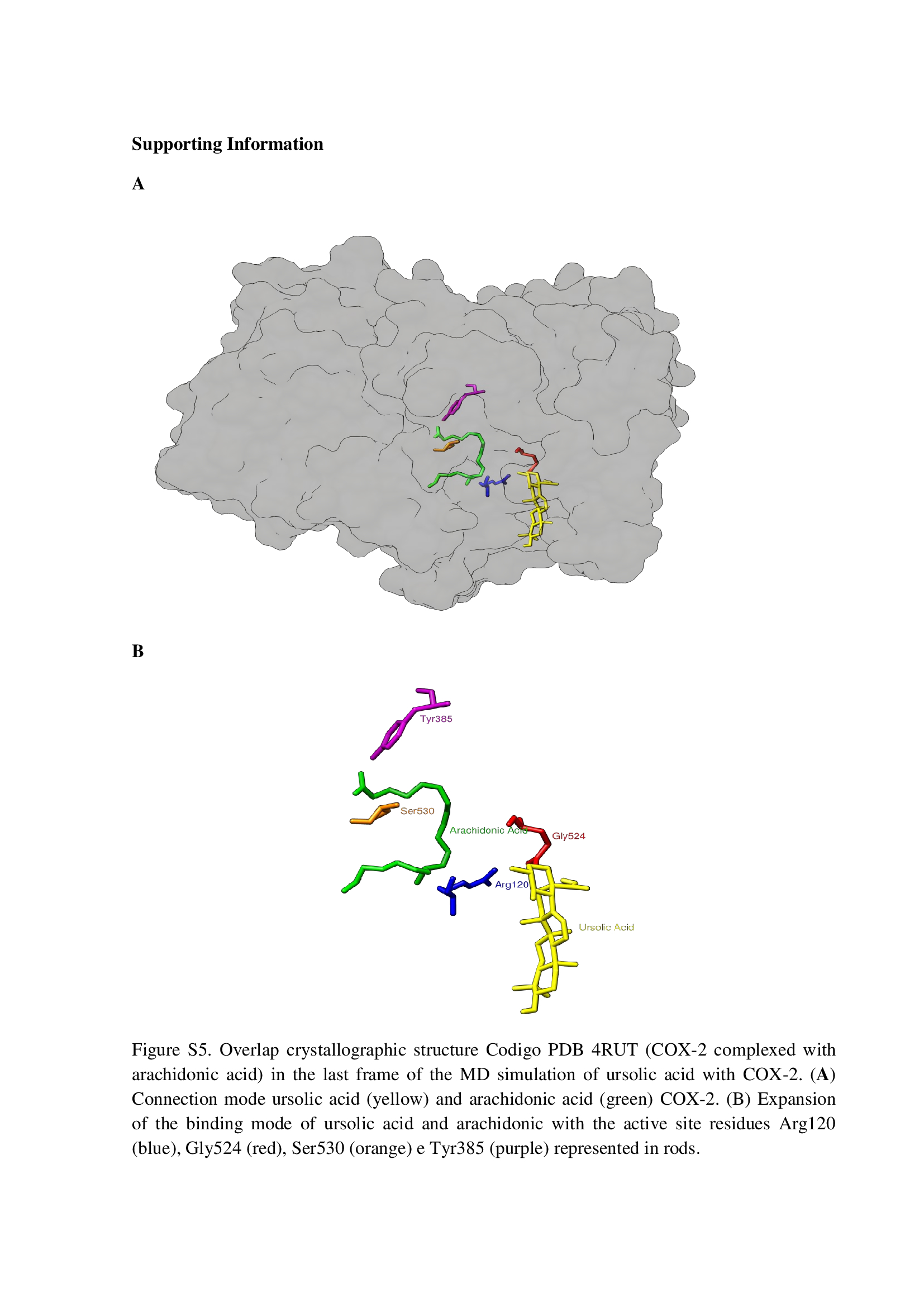

Supplement: Supplementary file 7 [file Image5.JPEG]
